# Influence of Supercritical Carbon Dioxide on the Activity and Conformational Changes of *α*-Amylase, Lipase, and Peroxidase in the Solid State Using White Wheat Flour as an Example

**DOI:** 10.3390/foods12244499

**Published:** 2023-12-16

**Authors:** Milena Ivanović, Željko Knez, Maja Leitgeb

**Affiliations:** 1 Faculty of Chemistry and Chemical Engineering, University of Maribor, Smetanova ulica 17, 2000 Maribor, Slovenia; milena.ivanovic.003@gmail.com (M.I.); zeljko.knez@um.si (Ž.K.); 2 Faculty of Medicine, University of Maribor, Taborska ulica 8, 2000 Maribor, Slovenia

**Keywords:** enzyme inactivation, supercritical carbon dioxide, α-amylase, lipase, horseradish peroxidase, structural changes, CD spectroscopy

## Abstract

Green technologies using renewable and alternative sources, including supercritical carbon dioxide (sc-CO_2_), are becoming a priority for researchers in a variety of fields, including the control of enzyme activity which, among other applications, is extremely important in the food industry. Namely, extending shelf life of e.g., flour could be reached by tuning the present enzymes activity. In this study, the effect of different sc-CO_2_ conditions such as temperature (35–50 °C), pressure (200 bar and 300 bar), and exposure time (1–6 h) on the inactivation and structural changes of *α*-amylase, lipase, and horseradish peroxidase (POD) from white wheat flour and native enzymes was investigated. The total protein (TPC) content and residual activities of the enzymes were determined by standard spectrophotometric methods, while the changes in the secondary structures of the enzymes were determined by circular dichroism spectrometry (CD). The present work is therefore concerned for the first time with the study of the stability and structural changes of the enzyme molecules dominant in white wheat flour under sc-CO_2_ conditions at different pressures and temperatures. In addition, the changes in aggregation or dissociation of the enzyme molecules were investigated based on the changes in particle size distribution and ζ-potential. The results of the activity assays showed a decrease in the activity of native POD and lipase under optimal exposure conditions (6 h and 50 °C; and 1 h and 50 °C) by 22% and 16%, respectively. In contrast, no significant changes were observed in *α*-amylase activity. Consequently, analysis of the CD spectra of POD and lipase confirmed a significant effect on secondary structure damage (changes in *α*-helix, *β*-sheet, and *β*-turn content), whereas the secondary structure of *α*-amylase retained its original configuration. Moreover, the changes in particle size distribution and ζ-potential showed a significant effect of sc-CO_2_ treatment on the aggregation and dissociation of the selected enzymes. The results of this study confirm that sc-CO_2_ technology can be effectively used as an environmentally friendly technology to control the activity of major flour enzymes by altering their structures.

## 1. Introduction

At a time when awareness of a healthy lifestyle is increasing, the production of fresh food with excellent nutritional properties is becoming a necessity. As a result, the use of fresh products on the market has increased by 30% from 2020 to today [1]. In most cases, however, it is not possible to meet the requirement that fresh food always be immediately available to consumers. Before reaching the final user, a raw food undergoes a long transportation and storage process that can lead to various quality changes, such as: (i) changes in the physical properties and appearance; (ii) changes in the nutritional properties; and finally, (iii) changes in the appearance, taste, and characteristics of the final products. This is a particular problem when food products are to be stored for a long period of time. One of these products is also flour. In the baking industry, enzymes including alpha-amylase, xylanases, lipases, oxidases, proteases and asparaginases are commonly used [2]. Each enzyme has a specific application in bread production. Enzymes are usually added to change the dough rheology, gas retention and softness of the crumb in bread making, to change the softness of the product and finally to reduce acrylamide formation in bakery products.

However, the uncontrolled activity of the enzyme lipase (EC 3.1.1.3) during storage leads to the degradation of lipids and the release of free fatty acids, which, combined with the undesirable activity of peroxidase (POD, EC 1.11.1.7) and the formation of aldehydes and ketones, results in a significant loss of its sensory properties and nutritional value, which in turn has negative effects on the human body [3]. Therefore, Lancelot et al. [4] recently proposed new guidelines for flour storage. They recommend storing flour at subzero temperatures in sealed containers for short storage periods, up to six months. However, for longer periods than six months, storage in paper containers at a temperature of −20 °C would be the best storage condition [4]. Another way to extend the shelf life of flour could be its pretreatment to inactivate enzymes and microorganisms, and storage without special temperature and humidity conditions. New research is therefore moving in the direction of finding a way to extend the shelf life of products while preserving their nutritional properties.

Traditional inactivation protocols for food enzymes include enzyme “roasting” [5,6], microwave irradiation [7,8] or superheated steam treatment [9]. However, all of these processes operate at significantly elevated temperatures, in most cases above 100 °C, resulting in serious destruction of the food structure and loss of its nutritional properties. To date, several innovative nonthermal technologies such as ultrasound [10,11], radiofrequency [12], electrospray [13], pulsed electric field [14], cold plasma [15], and supercritical carbon dioxide (sc-CO_2_) [16,17,18,19] have also been proposed for inactivation of unwanted enzymes in various foods. However, most of these papers focused on the inactivation of enzymes on foods in liquid and semi-liquid form. Only a limited number of recently published papers address the inactivation of enzymes in solid foods [3,20,21], while our research group is among the pioneers that have presented results on inactivation of enzymes from various flours using sc-CO_2_ technology [2,22,23].

The application of supercritical fluids as a non-thermal, anhydrous, environmentally friendly, economical, and clean technology has attracted great interest in the food industry in recent years. Among supercritical fluids, supercritical carbon dioxide (sc-CO_2_) is the most commonly used solvent, which has become a preferred solvent for various applications in the cosmetic, pharmaceutical, biomedical, and food industries [24,25,26,27]. Sc-CO_2_ is a fluid state of carbon dioxide (CO_2_) in which it is maintained at/above its critical temperature (31.1 °C) and pressure (73.8 bar). Due to its characteristic properties under these conditions (diffusion rates as in a gas and solvent densities as in a liquid), it is an ideal solvent for enzymatic reactions and offers the possibility of creating very environmentally friendly biotechnological applications [18]. However, the studies have also confirmed enzyme instability and short half-life of enzymes in sc-CO_2_ media, leading to complete denaturation of proteins in some cases [28,29]. As a result, the application of sc-CO_2_ in the inactivation of enzymes has attracted more and more attention of researchers in recent years. Most of the data published so far have examined the effect of sc-CO_2_ on enzymes belonging to the oxidoreductive subclass, such as POD, polyphenol oxidase (PPO), and lipoxygenase. For instance, one of the first papers describing the mechanism of inactivation of POD by sc-CO_2_ was published by Gui et al. [30]. They showed that the maximum inactivation of the buffer solution of POD occurs at a pressure of 300 bar and an elevated temperature of 55 °C, which leads to a decrease in the proportion of *α*-helix in the secondary structure of the enzyme. In addition, Marszałek et al. investigated the influence of sc-CO_2_ treatment on the inactivation of POD and POP from different fruit juices: beetroot [31], strawberry [32] and cloudy apple juice [17]. In general, they have demonstrated higher temperature and pressure resistance of PPO compared to POD under the influence of various sc-CO_2_ conditions. In the case of *α*-amylase (an enzyme belonging to the group of hydrolases), the enzyme activity was strongly dependent on sc-CO_2_ pressure and exposure time. Indeed, the highest activity (hyperactivation) of *α*-amylase was observed after 1 h at 300 bar and 35 °C (residual activity of 232%), while a decrease to 62% of initial activity was observed after prolonged exposure to sc-CO_2_ from 1 h to 24 h [33]. However, as with other innovative technologies, all of these papers focused on the effects of sc-CO_2_ conditions on enzyme activity in liquid and semi-liquid samples. Finally, some of the few articles analysing the effects of sc-CO_2_ on enzymes in the solid state are the works of Senyay-Oncel and Yesil-Celiktas [34] and Santos et al. [35] who studied the inactivation ratio of *α*-amylase and immobilised lipase, respectively, under sc-CO_2_ conditions. However, a deeper understanding of the changes that occur when the enzymes in the solid state are exposed to the influence of supercritical conditions is not described in these papers.

Therefore, there is currently insufficient information on the changes in enzyme structure that occur when solid foods are processed with sc-CO_2_, which raises new questions that need to be answered. In this work, we have attempted to answer some of these questions for the first time. The main objectives of this study were: (i) to analyse the influence of different supercritical conditions (temperature from 35 °C to 50 °C and pressure from 200 bar and 300 bar) on selected enzyme activities in white wheat flour; (ii) to determine the influence of sc-CO_2_ treatment on TPC in white wheat flour; (iii) determination of changes in the activity of native enzymes in the solid state (*α*-amylase, lipase and POD) before and after sc-CO_2_ treatment under different conditions; and (iv) evaluation of changes in the secondary and tertiary protein structures of native enzymes after sc-CO_2_ treatment.

## 2. Materials and Methods

### 2.1. Chemicals and Samples

The white wheat flour used in this study (milled in 2023) was kindly obtained from Hlebček d.o.o., Pragersko, Slovenia. Native enzymes were purchased as follows: *α*-amylase (~30 U/mg) from *Asperigillus oryzae* was purchased from Sigma, lipase (~200 U/mg) from *Asperigillus niger* from CioChemics, and horseradish peroxidase (POD, ~52 U/mg) from BBI Enzymes (Blaenavon, UK). Carbon dioxide (CO_2_, 2.5) used for the exposure experiments was purchased by Messer, (Ruše Slovenia). Ethanol (96%), phosphoric acid (≥85%), sodium chloride (≥99.5%), Coomassie-Brilliant Blue G250 and acetonitrile (99.9%) were supplied by Merck (Darmstadt, Germany), whereas bovine albumin serum (BSA) (≥96%), sodium acetate (≥99.0%), acetic acid (GR for analysis), p-nitrophenyl butyrate (≥98%) were supplied by Sigma Aldrich (St. Louis, MO, USA). All other chemicals used during the experiments were of analytical grade. Ultrapure water was prepared fresh daily in the laboratory.

### 2.2. Supercritical Carbon Dioxide Treatment

Sc-CO_2_ treatment was performed according to a method described in a recent paper of our research group, with some modification [2]. Briefly, 5 g of white wheat flour packed in a filter bag was placed in a high-pressure batch reactor equipped with a 60 mL high-pressure vessel, heating and temperature control modules, pressure control valves, flow meters, and safety devices. After reaching the specified experimental conditions for pressure (200 bar or 300 bar) and temperature (35 °C, 42.5 °C or 50 °C), the samples were left in the high-pressure reactor for a well-defined time of 3 h. The experimental conditions for pressure and temperature were chosen based on the results of our previous studies and the investigation of the feasibility and economic viability of the process itself. In order to avoid temperature-related inactivation of the enzymes, the upper temperature limit was set at 50 °C. Rapid depressurization followed, and the flour samples were immediately used for extraction of total proteins and determination of residual enzyme activities.

To determine the effect of sc-CO_2_ treatment on the conformational changes of the enzymes studied, native *α*-amylase, lipase, and POD were exposed to sc-CO_2_ in the solid state under the specific conditions determined in the preliminary experiments on the flour samples. Indeed, these experiments were performed at two different temperatures (35 °C and 50 °C), at a constant pressure of 300 bar, and for different time periods from 1 h to 6 h.

### 2.3. Extraction Protocol and Determination of Total Protein Content

Extraction of proteins from untreated and sc-CO_2_ treated flour samples was performed according to the protocol fully optimised in the study by Hojnik Podrepšek et al. [36]. Briefly, 5 g of the selected flour sample and five glass beads were placed in a conical Erlenmeyer flask. Then, 30 mL of the 0.1 M acetic buffer (pH = 5.3) was added. The Erlenmeyer flask, sealed with parafilm, was shaken at 300 rpm for 90 min at a controlled temperature of 25 °C. To obtain a clear supernatant, the suspension was centrifuged at 8000 rpm for 5 min at room temperature (RT, 25 °C).

TPC was determined by the standard Bradford spectrophotometric method. BSA as the standard protein was used to generate the calibration curve ranging from 0.1 mg/mL to 1 mg/mL. In this assay, 20 µL of BSA or appropriately diluted flour extract was added to 980 µL of Bradford reagent. After incubation of the reaction mixture for 15 min at RT, absorbance was measured with a UV spectrophotometer (Varian Cary Probe 50, Agilent Technologies, Santa Clara, CA, USA) at 595 nm using the Bradford reagent with 20 µL ultrapure water as a blank. TPC was determined from the calibration curve, and results were expressed as mg BSA per mL sample (mg/mL).

### 2.4. Determination of Enzymes Residual Activities

The activities of specific enzymes from untreated and sc-CO_2_ treated flour samples and native enzymes were determined according to standard activity protocols using a UV spectrophotometer (Varian Cary Probe 50, Agilent Technologies, Santa Clara, CA, USA).

The *α*-amylase activity was determined by the DNS method, previously described, with some modifications [11]. Briefly, for DNS reagent preparation, 1 g of 3,5-dinitrosalicylic acid (DNS) was dissolved in 50 mL of ultrapure water and 20 mL of 2 M NaOH. Then, 30 g of potassium sodium tartrate (KNaC_4_H_4_O_6_ × 4H_2_O) was added, and the reagent was diluted with ultrapure water to a final volume of 100 mL. In addition, a 1% starch solution (from wheat) was prepared in 0.02 M sodium phosphate buffer containing 0.006 M NaCl. The basic principle of this method is based on the conversion of starch to maltose by *α*-amylase, the content of which was measured by reduction of DNS. Therefore, to prepare the calibration curve, standard solutions of maltose were prepared in a concentration range from 0.5 mM to 5 mM. A reaction mixture containing 0.5 mL of properly diluted untreated, and sc-CO_2_ treated samples of flour or native enzymes/maltose solution/ultrapure water (as blank) and 0.5 mL of starch solution was incubated for 3 min at RT. In addition, 1 mL of DNS reagent was added, and the mixture was boiled in a water bath for 5 min. After cooling to RT, 10 mL ultrapure water was added and the absorbance of the blank solution, samples, and maltose standards was measured at 540 nm using the spectrophotometer.

Lipase activity was determined spectrophotometrically using p-nitrophenyl butyrate (*p*-NPB) as substrate [37]. For this assay, the solution of the untreated and sc-CO_2_ treated flour extracts was used without further dilution, while the untreated and sc-CO_2_ treated native lipase was prepared at a concentration of 10 mg/mL. The reaction mixture of 900 µL sodium phosphate buffer (PBS, pH = 7.2) containing 150 mM sodium chloride and 0.5% Triton X100 (*v*:*v*) and 100 µL flour samples/enzyme solutions or phosphate buffer (as blank) was incubated for 3 min at 37 °C. Subsequently, 10 µL of p-NPB (50 mM in acetonitrile) was added and the release of the NPB anion was monitored at 400 nm for 5 min.

The activity of POD was determined by the standard spectrophotometric method using 4-aminoantipyrine (4-AAP) as a substrate in the presence of hydrogen peroxide (H_2_O_2_), described in the previously published work of our research group [22]. Briefly, a reaction mixture containing 1.4 mL of 4-AAP (2.5 mM, with 0.17 mM phenol), 1.5 mL of H_2_O_2_ (1.7 mM in 0.2 M PBS), and 0.1 mL of PBS was prepared and 0.5 mL of properly diluted enzyme solution (untreated or sc-CO_2_ treated flour extracts or native enzymes) was added. A blank sample was prepared such that 0.5 mL of PBS was added instead of 0.5 mL of the sample. The change in absorbance was measured immediately after addition of the sample at 510 nm for 4 min using a spectrophotometer.

The residual activity for all investigated enzymes was determined by the following equation:

(1) $$Residual\;activity\;\left[RA;\;\%\right]=\frac{\frac{U}{mg}(sc-{CO}_{2}\;treated\;enzyme)}{\frac{U}{mg}(untreated\;enzyme)}\times 100$$

### 2.5. Circular Dichroism Spectroscopy

CD the spectra of the native enzymes (1 mg/mL in ultrapure water for *α*-amylase and lipase and PBS buffer for POD) were recorded in the far UV region (190–260 nm) at RT using a spectrometer (JASCO Corp., Tokyo, Japan) under continuous nitrogen flow with a quartz cuvette with 1 mm path length. The bandwidth was 1 nm, and the scan speed was 100 nm/min, with three replications for each spectral line [38]. All the spectra were corrected by subtracting the baseline. The secondary structure composition of the enzymes analyzed, expressed in the percentages (%), was calculated using SpectraMenager Software version 10.0 from JASCO.

### 2.6. Determination of Zeta Potential

Zeta potential analysis was conducted by using Zetasizer Nano ZS instrument (Malvern Instruments Co., Malvern, UK) through dynamic light scattering. All measurements were performed at RT with an equilibrium time of 120 s.

### 2.7. Determination of Particle Size Distribution

All samples were dissolved in ultra-pure water at the appropriate concentrations. Measurements were performed with the Zetasizer Nano ZS (Malvern Instruments Co., Malvern, UK) using 10 mm polystyrene cuvettes at RT and an equilibration time of 120 s. The particle size distribution of the enzyme solutions (before and after sc-CO_2_ treatment) was expressed in nm based on the fractional intensities.

### 2.8. Statistical Analysis

All experiments in this study were performed in duplicate, whereas instrumental measurements were performed in triplicate. Results were expressed as mean ± standard deviation. An analysis of variance (ANOVA) followed by a Student–Newman–Keuls (S-N-K) post-hoc test was performed to examine a difference between samples at the probability level of *p* < 0.05 using IMB SPSSS (version 21.0, Chicago, IL, USA). All figures were created using Microsoft Office Excel version 2311.

## 3. Results and Discussion

### 3.1. Inactivation Rate of α-amylase, Lipase, and POD in White Wheat Flour

To date, sc-CO_2_ technology has been effectively used for food preservation and enzyme activity control, especially for PPO inactivation in liquid and semi-liquid foods [19,24,39], due to its proven advantages over traditionally used thermal methods. However, there are only a limited number of papers investigating the effect of sc-CO_2_ on pasteurization of solid samples [40,41], while our research group was the pioneer that opened new possibilities for the use of this technology to extend the storage time of flour and improve its psychochemical properties [2,23,36]. This is of crucial importance for the bakery industry, as the results of the recent study published by Lancelot et al. showed that long-term storage of flour at room temperature leads to a significant change in its parameters and baking properties [4]. The present work is therefore concerned for the first time with the study of the stability and structural changes of the enzyme molecules dominant in white wheat flour under sc-CO_2_ conditions at different pressures and elevated temperatures (42.5 °C and 50 °C).

The results of determination of total proteins in flour sample and changes in selected enzyme activities before and after sc-CO_2_ treatment at three different temperatures (35 °C, 42.5 °C and 50 °C) and two different pressures (200 bar and 300 bar) for 3 h are presented in Table 1. As can be seen from the results, no significant changes in total protein content were observed in the samples after sc-CO_2_ treatment compared to the untreated flour, regardless of pressure and temperature, so the nutritional composition of the flour was not affected. This is extremely important, as the protein content determines the specific use of the flour and contributes to the desired taste and texture of the bread and other bakery products. For comparison, Bahrami et al. also reported that no changes in total proteins were detected after cold plasma treatment of white flour, although a significant change in molecular structures and organizations was confirmed [42].

Checking the studied enzymes activities, no common rule could be set to describe the influence of the treatment parameters. For α-amylase activity, i.e., almost no negative changes in enzyme activity were detected compared with untreated sample. Although physical parameters such as density and dielectric constant depend on the pressure and temperature of the supercritical fluid, and an increase in pressure increases the solubility and thus the dissolving power of these solvents [34], the treatments under the conditions used in our study apparently had no significant effect on the activity of *α*-amylase in the solid state. The highest increase in this enzyme activity was achieved at 200 bar and 42.5 °C with the activity rise of 25.0%. Those results agreed well with those obtained by Yadav and Prakash, who studied the activity and thermal stability of *α*-amylase, where the optimal temperature for the enzyme was 50 ± 2 °C [43]. In another example, the thermal stability of *α*-amylase was demonstrated even up to 70 °C [44].

On the other hand, the initial activity of lipase was strongly affected by the influence of sc-CO_2_ treatment, reaching the lowest value at a temperature of 50 °C and a pressure of 300 bar (the residual activity was only 18.4 ± 1.2%). These results agree well with the study of Mathias et al. who showed that both factors, CO_2_ pressure and temperature, affect lipase inactivation [45]. In their study, there was a complete loss of lipase activity in the buffer with a pH of 5.6 after exposure to the supercritical conditions of 250 bar and 60 °C for 30 min. It is also important to emphasize that the percentage of lipase inactivation in our study with the use of supercritical CO_2_ was higher than the results for lipase inactivation (inactivation rate of 50%) from barley flour recently presented by Li et al. [3], and correlates well with the results of Ling at al. who evaluated the inactivation of lipase from rice bran (retained activity of 19.2%) [46]. However, both studies used heat-assisted enzyme inactivation pretreatments at a temperature above 100 °C, which affects the physicochemical properties of the samples, which can be successfully avoided by using the sc-CO_2_ technology proposed in our study.

In the case of POD, an increase in activity was observed at a temperature of 35 °C compared to the untreated samples at both applied pressures, with the higher value obtained at 300 bar (35% increase in activity) (Table 1). However, with increasing temperature (50 °C), the initial enzyme activity was preserved. Thus, it is of great importance to understand the mechanism leading to the changes of enzyme activities under the influence of supercritical conditions. This phenomenon can probably be explained by the different moisture content of the samples [47], because it was found out in the early studies [48] and was confirmed lately [49], that water activity has a strong influence on enzyme activity in sc-CO_2_. According to this, the influence of sc-CO_2_ in the presence of a large amount of water is associated with the formation of carboxylic acid, which leads to a decrease in the pH of the sample in the reactor, resulting in the disruption of the protein structure and finally inactivation/hyperactivation of the enzymes [50]. This is one of the possible theories of how a change in enzyme activity occurs under supercritical conditions. In addition, Li et al. assume that small molecules (CO_2_, H_2_CO_3_, HCO_3_^−^ and CO_3_^2−^) block the access of substrates to the active site of phenylalanine ammonia lyase, leading to a decrease in its activity [38]. In contrast, older theories assume that the destabilization of protein molecules in sc-CO_2_ is mainly due to the role of lysine residues on the molecular surface [51]. This theory hypothesizes that the uncharged lysine side chain reacts with CO_2_, resulting in the formation of carbamate, a carbamic acid-derived organic compound that adversely affects the reaction catalysed by the enzyme and eventually leads to its inactivation. Finally, Monhemi and Jalali recently presented a new theory for the denaturation of enzymes under CO_2_ conditions based on molecular docking analysis [18,28]. According to these authors, the denaturation process of proteins is not limited to lysine, but all surface-charged and polar residues can play an essential role in the destabilization of enzymes under supercritical conditions. Namely, they found that the formation of new non-native interactions of charged and polar residues on the enzyme surface led to the reduction of some important native interactions through the formation of new H-bonds. Consequently, destabilization and denaturation of the enzymes occurred in sc-CO_2_. It is also worth noting that the stability and activity of enzymes under supercritical conditions is highly dependent on many other factors, including: enzyme species, exposure time, pressure, temperature, and operating mode of the reaction system [49,52,53]. To gain a deeper understanding of the structural changes of enzymes in solid matrix exposed to supercritical conditions, we continued our study on native enzymes predominant in white flour: *α*-amylase, lipase, and POD. For these studies, we chose the conditions that induce the strongest changes in flour enzymes, i.e., a pressure of 300 bar and two different temperatures (35 °C and 50 °C), while the exposure time ranged from 1 h to 6 h.

### 3.2. Effect of sc-CO_2_ Treatment on the Native Enzyme Activity

**Figure 1:**
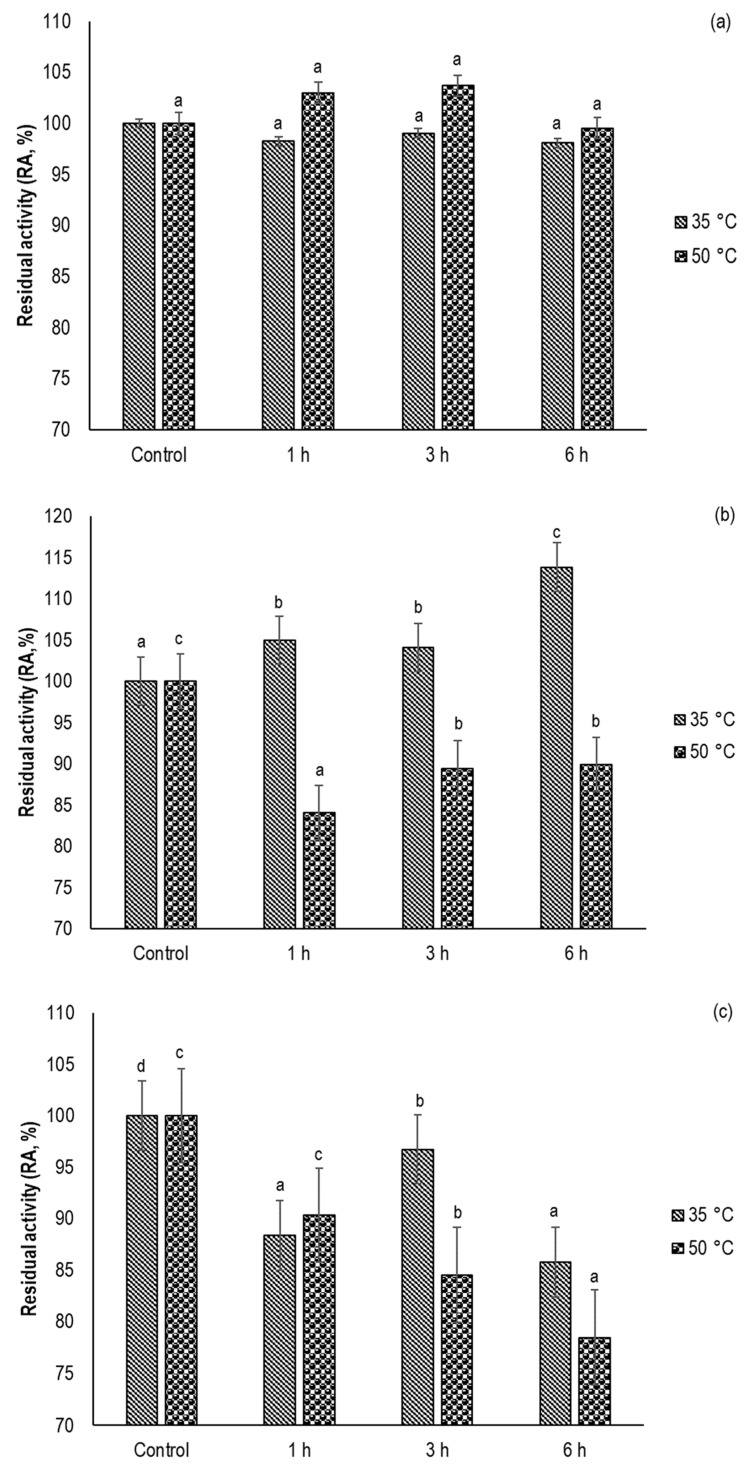
Effect of sc-CO_2_ on the activity of native enzymes: (**a**) *α*-amylase, (**b**) lipase, and (**c**) POD at the temperatures 35 °C and 50 °C for different time periods (1–6 h). The pressure was 300 bar. ^a,b,c,d^ Different superscripts for the samples at the same temperature indicate significant differences between the values obtained at the 95% confidence level (*p* < 0.05) according to the S-N-K test.

The effect of sc-CO_2_ treatment on the activity of native *α*-amylase, lipase, and POD at two different temperatures (35 °C and 50 °C), at a constant pressure of 300 bar, and as a function of exposure time (1–6 h) is shown in Figure 1a–c, respectively. As evident from Figure 1a, no negative activity changes were observed for *α*-amylase under the conditions tested, which is consistent with the results obtained for the white wheat flour sample. These results are also in good agreement with a previously published study by Senyay-Oncel and Yesil-Celiktas, who showed that sc-CO_2_ with a pressure below 300 bar did not affect the stability of *α*-amylase in the solid state [34]. Moreover, in the study by Senyay-Oncel and Yesil-Celiktas a statistically significant decrease in enzyme activity at a temperature of 55 °C was demonstrated, while a further increase in temperature led to a re-increase in *α*-amylase activity. Similarly, a recent study monitoring the stability of *α*- and *β*-amylase with increasing temperature during the mashing process of barley malt showed significantly higher stability of *α*-amylase compared to its conformational *β*-isomer [44]. Indeed, in this study, no statistically significant change in *α*-amylase activity was observed in the temperature interval from 20 °C to 70 °C, and the critical temperature for alerting enzyme activity was set at 72.5 °C. On the other hand, high sensitivity of *α*-amylase to the influence of ultrasound was demonstrated, with the highest reported inactivation rate observed during 60 min of ultrasound irradiation, which preserved only 17% of the original enzyme activity [11].

In the case of lipase, a strong correlation can be seen between the changes in enzyme activity with increasing temperature and exposure time (Figure 1b). Thus, at a temperature of 35 °C, an increase in lipase activity with increasing exposure time could be observed, up to a maximum of 115% of the initial activity determination after 6 h of exposure to sc-CO_2_. On the other hand, the initial lipase activity decreased at 50 °C, with the lowest value determined after 1 h of the sample exposed to the supercritical conditions.

The inactivation and determined residual activity of crude POD, treated with sc-CO_2_ at a constant pressure of 300 bar at two temperatures (35 °C and 50 °C), for different time periods (1–6 h), is shown in Figure 1c. As can be seen from the figure, the stability of POD was affected by both, increasing the temperature and extending the exposure time. The highest inactivation level was obtained after 6 h of sc-CO_2_ treatment at 50 °C. These results can be probably explained by the fact that the free energy (∆G) of proteins is highest at 37 °C; the protein is in the native state. As the temperature increases, an equilibrium between the unfolded and native states is established, while as the temperature continues to rise, the denaturation process occurs spontaneously (∆G < 0) [12]. The inactivation rate of POD in our study agrees well with previously published studies in which the authors showed that the percentage of POD inactivation is directly related to the increase in temperature, pressure, and duration of sc-CO_2_ treatment [16,17,19,30]. However, a direct comparison of the results with the literature is not possible because this is the first time that our research group has worked with the native POD subjected to the denaturation process in the solid state, whereas previous studies were performed with enzyme buffer solutions or liquid and semi-liquid foods. For example, in the study by Zambon et al. the inactivation of total POD and PPO in strawberries was observed at a temperature of 40 °C and a pressure of 133 bar during a 7 h sc-CO_2_ treatment [19], proving a very successful application of supercritical technology with the use of CO_2_ for food pasteurization.

Finally, the disagreement in the percentage of lipase and POD inactivation compared to the flour samples discussed in the previous section is likely due to two main factors. First, as explained earlier, the effect of supercritical conditions on the stability of the enzymes depends strongly on the water content. Indeed, in the experiments, anhydrous enzymes were used in the solid state, while the moisture content of the flour samples is usually between 9–15% [23]. Another very important factor is the exposure of the enzyme to the conditions studied. The enzymes in flour are located in an aleurone layer above the endosperm of the grain and are therefore more difficult to access compared to native enzymes. The protein content of the flour sample is extremely important for the final result since it is in direct connection with CO_2_ solubility [23] and consequently pH change or chelation. In various flour types it differs and so does the CO_2_ solubility. The before-mentioned changes lead to different results about enzyme inactivation.which also depends on the moisture content of the sample. Therefore, there is sometimed hard to achieve the reproducibility of the results. This could be one of the obstacles when using supercritical fluid technology as the method for enzyme inactivation in flour.

To study the effects of the exposure of the enzymes to supercritical conditions on their structure, further analyses were performed to evaluate the changes in the tertiary and secondary structure of the proteins.

### 3.3. Effect of sc-CO_2_ on the Secondary Structure of the Investigated Enzymes

**Figure 2:**
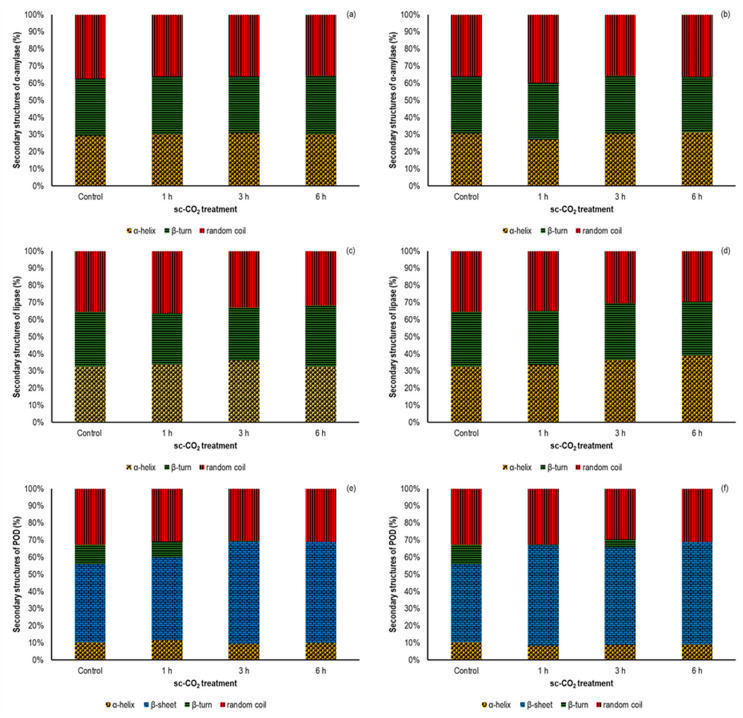
Secondary structure content (%) of *α*-amylase, lipase, and POD before and after sc-CO_2_ treatment for the different exposure times (1 h, 3 h, and 6 h) and 300 bar; (**a**) *α*-amylase at 35 °C; (**b**) *α*-amylase at 50 °C; (**c**) lipase at 35 °C; (**d**) lipase at 50 °C; (**e**) POD and 35 °C; and (**f**) POD at 50 °C.

**Figure 3:**
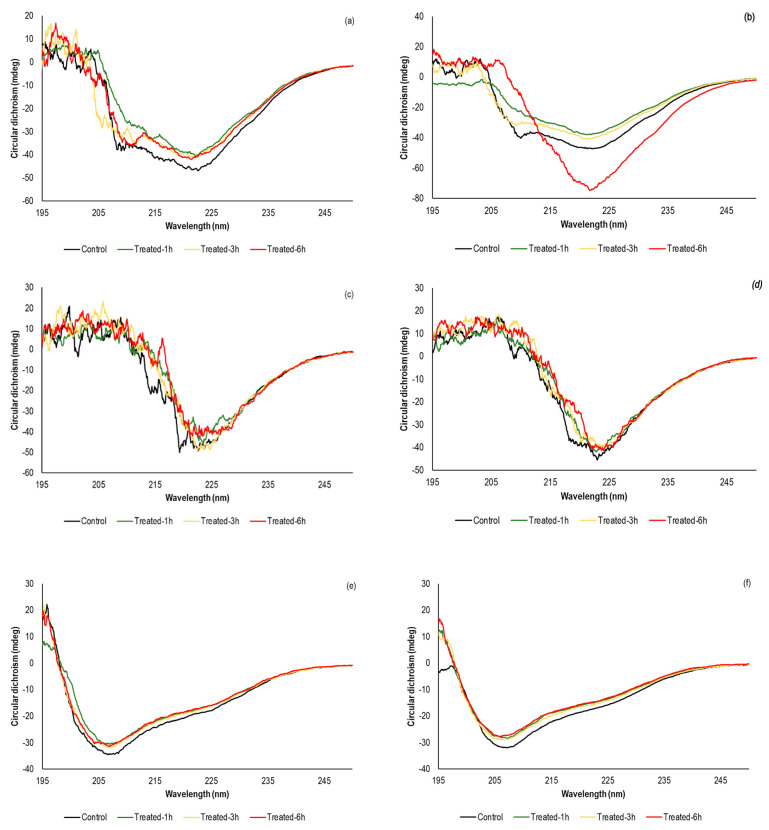
Circular dichroism (CD) spectrum of native and sc-CO_2_ treated *α*-amylase, lipase, and POD, at 300 bar, for different time periods (1–6 h). (**a**,**b**) *α*-amylase at 35 °C and 50 °C, respectively; (**c**,**d**) lipase at 35 °C and 50 °C, respectively and (**e**,**f**) POD at 35 °C and 50 °C, respectively.

CD spectroscopy is the most commonly used instrumental technique for the study of protein secondary structures, in which the content of *α*-helix, *β*-sheet, *β*-turn, and random coil is estimated from changes in the intensity and shape of the peaks in the CD spectra [12,54,55]. Based on the position of the peak maximum on the positive and negative sides of the CD spectra, the secondary structure of the protein can be evaluated. For example, the proteins with a high proportion of *α*-helix in the structure have negative bands at 222 nm and 208 nm and the positive band at 193 nm, while the well-defined antiparallel *β*-sheets have negative bands at 218 nm and positive bands at 195 nm [56]. Figure 2a–f show the percentage of *α*-helix, *β*-sheet, *β*-turn, and random coil in the secondary structures of *α*-amylase, lipase, and POD before and after sc-CO_2_ treatment, while the appropriated CD spectra are shown in Figure 3a–f.

The CD spectra of *α*-amylase (Figure 3a,b) showed that the initial secondary structure contained 29.3% *α*-helix, 33.3% *β*-turn, and 37.4% random coil after fitting the curve with the Young’s model (Figure 2a,b), which agreed well with literature data [57]. When the enzyme was treated with sc-CO_2_ at 35 °C, the amount of *α*-helix (30.3%, 30.6%, and 30.2% after 1 h, 3 h, and 6 h, respectively) and *β*-turn (33.7%, 33.3%, and 34.1% after 1 h, 3 h, and 6 h, respectively) increased, while the content of random coil (36.0%, 36.1%, and 35.7% after 1 h, 3 h, and 6 h, respectively) decreased. However, these changes were not statistically significant and agreed well with the enzyme activity results discussed previously. Also, in the recently published study by Li et al. who studied *α*-amylase activity under the influence of pulsed electric field, the authors showed a high stability of the sample after an initial exposure cycle, in which the original secondary structure of the protein was preserved [57]. On the other hand, Tian et al. reported a positive effect of pulsed electric field on *α*-amylase activity, reaching the maximum at an electric field strength of 15 kV/cm and a flow rate of 100 mL/min, where a 22% increase in initial activity was obtained [58]. These results correlated with the increase in *α*-helix and *β*-turn content and a decrease in the *β*-sheet and random coil in the secondary structure of the enzyme. In our study, similar results were obtained when the samples were exposed to an elevated temperature of 50 °C. Indeed, the slight increase in *α*-amylase activity after 1 h and 3 h of sc-CO_2_ treatment was followed by an increase in *α*-helix content (up to 31.6%) due to a decrease in random coil content (Figure 2b). However, since it was previously shown that *α*-amylase activity remained unaffected, it can be concluded that these changes in the secondary structure of the enzyme are not sufficient to affect its activity.

The changes in the secondary structure of lipase before and after treatment with sc-CO_2_ are shown graphically in Figure 2c,d. Surprisingly, when we compare these results with the lipase activity results, we find a slight increase in *α*-helix content and a decrease in *β*-turn content and random coil. Comparing these results with data from the literature, a significant deviation from the trend can be also observed [59,60,61]. For example, Esmaeilnejad-Ahranjani et al. reported changes in lipase secondary structure after immobilization of the enzyme on polyeletrolyte-coated magnetic silica nanocomposite particles and found that the loss of enzyme activity was accompanied by a decrease in the content of *α*-helix and *β*-sheets and an increase in the content of disordered elements [59].

In the case of POD, the intense negative peaks were observed at 208 nm and 226 nm (Figure 3e,f), which was in consistent with the observation of Xu et al. [13], while the secondary structure of the protein showed a content of 10.3% *α*-helix, 45.6% *β*-sheet, 11.3% *β*-turn, and 32.8% random coil. As shown in Figure 3e,f, compared to untreated POD, an up-shift in these peaks was observed at both investigated temperatures, and the intensity of the changes varied with exposure time, indicating the changes in the secondary structure of the enzyme. Consequently, a decrease in the content of the *α*-helix and an increase in the *β*-turn were observed under the influence of sc-CO_2_ (Figure 2e,f). When the temperature of the high-pressure reactor reached 50 °C, the contents of *α*-helix after sc-CO_2_ treatment were 8.4%, 8.7%, and 9.1% for 1 h, 3 h, and 6 h, respectively, significantly lower than those of the nontreated sample. At the same time, an increase in *β*-sheet content from an initial 45.6% to 58.6%, 57.1%, and 59.8% was observed during the 1 h, 3 h, and 6 h sc-CO_2_ exposures, respectively. Considering that the secondary protein structure is stabilized by various hydrogen bonds [12,46], it can be predicted that exposure to CO_2_ under high pressure in combination with elevated temperature can break some of these bonds, converting an ordered helical structure into a disordered structure. Similar observation was pointed out in the recently published papers, in which the other non-thermal techniques were applied to inactivate of POD. For instance, Yao et al. have investigated the influence of radio frequency heating in the temperature range from 50 °C to 90 °C at different electrode gaps (100 nm, 110 nm and 120 nm) [12]. They found that long time-low heating rate RF heating at the largest electrode gap promote significant structural changes in the enzyme secondary structure. On the other hand, in recently published study by Guo et al., it was found that *α*-helix content was mostly reduced in the dual-frequency mode; while the temperature showed less influence in the POD inactivation at the same frequency applied [62]. In the case of when the POD was treated by 80 W ultrasound, a corresponding CD spectra did not show a changes in the negative peaks position, while the content of *α*-helix was decreased from initial 35.32% to 30.94% on account of *β*-sheet and random coil increase from 5.37% to 9.42% and from 29.56% to 33.07%, respectively [63]. The results of our study as well as those of the previously published data unquestionably show the critical relationship between content of *α*-helix and *β*-turn and activity of the POD.

### 3.4. Influence of sc-CO_2_ Treatment on the Particle Size Distribution

**Figure 4:**
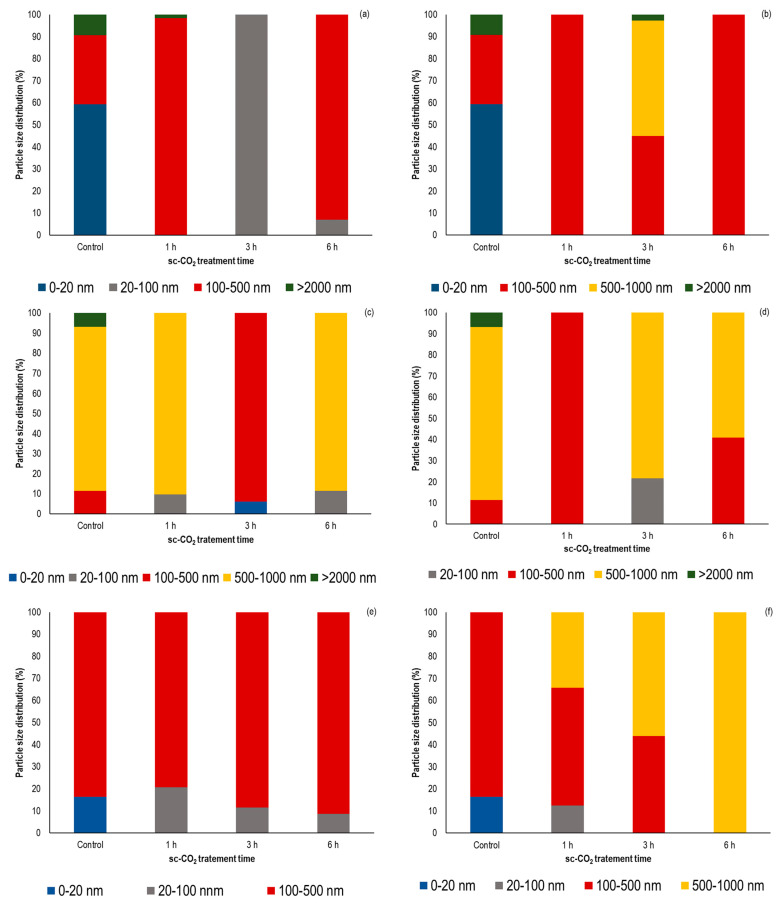
Particle size distribution (%) *α*-amylase, lipase, and POD before and after sc-CO_2_ treatment for the different exposure times (1 h, 3 h, and 6 h) and 300 bar; (**a**) *α*-amylase at 35 °C; (**b**) *α*-amylase at 50 °C; (**c**) lipase at 35 °C; (**d**) lipase at 50 °C; (**e**) POD et 35 °C and (**f**) POD at 50 °C.

As known from the literature, sc-CO_2_ treatment leads to different homogenization and aggregation effects in the treated enzyme solutions, resulting in changes in the particle size distribution of enzyme molecules [38,64]. However, in our study, we investigated the effects of supercritical conditions on the particle size distribution of the native enzymes in the solid state for the first time, and the results are shown graphically in Figure 4a–f. To facilitate interpretation of the results, all particle sizes are divided into well-defined size ranges: 0–20 nm, 20–100 nm, 100–500 nm, 500–1000 nm, and particle size larger than 2000 nm.

As shown in Figure 4a,b, the initial mean particle size of *α*-amylase powder was predominantly within 10 nm (59.4%), which correlates well with previously published literature data [57]. The particle size distribution of *α*-amylase was obviously affected by sc-CO_2_ treatment at different temperatures and different exposure times. In fact, only particles with the size between 100 nm and 500 nm (221 nm and 266 nm at 35 °C and 50 C, respectively) were observed after 1 h of sc-CO_2_ treatment at both temperatures studied. Further extension of the exposure time resulted in differences in the particle size distribution as a function of temperature. At 35 °C, a disaggregation appeared to a particle size from 20 nm to 100 nm, which coincides with the slight *α*-amylase activity increase (+15.2%) at these conditions (Table 1). Unexpectable, increasing the temperature promoted further aggregation of the particles, resulting in the appearance of a fraction of particles with a size of 500–1000 nm with a proportion of 52.4%, being one of the reasons for a slight activity drop to 112.1% (Table 1) of this enzyme. After a 6 h treatment, the results showed the same distribution of *α*-amylase particles as in the example of a 1 h treatment.

The particle size distribution of untreated lipase showed the presence of three particle size ranges with different contents. Most of the particles (~82%) were ~750 nm in diameter, while the fraction of smaller particle diameter (107 nm) was ~11% and the fraction of large diameter (5277 nm) was ~7% (Figure 4c,d). When the samples were exposed to sc-CO_2_ for 1 h at 35 °C, a decrease in particle size distribution could be observed (Figure 4c). This decrease was even more extreme when the experimental temperature was 50 °C (Figure 4d), when the only identified section of particles diameter was 318 nm. A further increase in exposure time at a temperature of 35 °C led to a further decrease in the particle’s diameter of the enzyme powder, while at a temperature of 50 °C a reaggregation occurred. This trend of particles aggregation continued further with increasing exposure time. Comparing these results with the results of the activity tests, the general conclusion can be drawn that the lowest activity was found at both temperatures tested when the particle’s diameter was in the range of 500–1000 nm. Similarly, Nadar and Rathod have found that the aggregation of lipase exposure to the continuous ultrasound resulted into decrease in the initial enzyme activity [65].

Finally, the particle size distribution patterns of POD before and after sc-CO_2_ treatment for the different time periods and two temperatures studied (35 °C and 50 °C) are shown in Figure 4e,f. As can be seen from the figures, the particle size distribution of the untreated POD was mainly composed of two particle size sections. The first section contained particles with a mean size of about 17 nm (16%), while the second section consisted of particles with a larger diameter, about 390 nm with an intensity of 84%. In general, exposure of POD to sc-CO_2_ conditions lead to aggregation of POD powder particles into clusters with larger diameter. At an experimental temperature of 35 °C, the proportion of enzyme molecules with a diameter of 100–500 nm increased linearly, while the intensity of molecules with a particle diameter below 100 nm decreases. Moreover, further increase of temperature up to 50 °C lead to further agglomeration of enzyme powder, resulting in the appearance of diameter greater than 500 nm, which linearly increased with increasing exposure time. Consequently, when the enzymes were exposed to sc-CO_2_ for 6 h, particle size of 577 nm were detected in the powder sample. This aggregation of POD probably affected some catalytic enzyme sites, leading to a decrease in the original enzyme activity. The results of our study were in good agreement with previously published data by Xu et al. [13]. Namely, they observed that the increase in particle size POD after electrospray treatment could lead to inactivation of the enzyme. In addition, the study by Li et al. confirmed that the exposure of phenylalanine ammonia lyase to sc-CO_2_ conditions leads to depolymerization at a pressure of less than 200 bar, while enzyme aggregates are formed at a pressure of more than 200 bar [38].

### 3.5. Influence of sc-CO_2_ Treatment on the ζ-Potential

The ζ-potential reflects the magnitude of electrostatic interactions between charged molecules in solutions, and its absolute magnitude indicates the magnitude of electrostatic forces between particles [38]. It is a generally accepted rule that particles with an absolute ζ-potential value of 30 mV can be considered stable. Therefore, measurement of changes in ζ-potential of enzymes can also be used to monitor changes on their molecular surfaces and consequently the influence of these changes on enzyme activities. Therefore, in this work, we also investigated the change in ζ-potential of *α*-amylase, lipase, and POD after sc-CO_2_ treatment. The raw data obtained are shown in the Supplementary Materials (Figures S1–S3).

**Figure 5:**
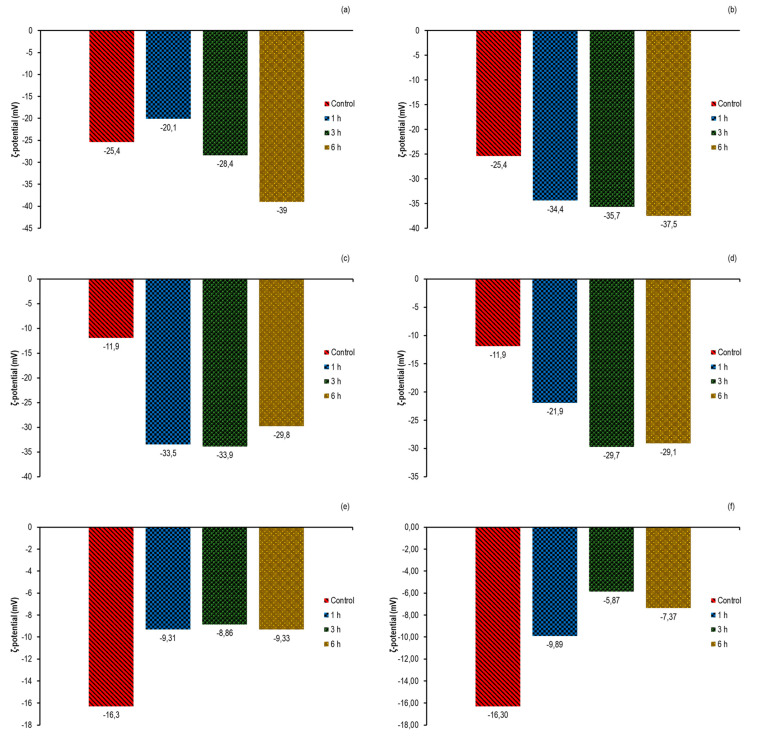
ζ-potential of the studied enzymes after sc-CO_2_ treatment as a function of exposure time at 300 bar. (**a**) *α*-amylase at 35 °C; (**b**) *α*-amylase at 50 °C; (**c**) lipase at 35 °C; (**d**) lipase at 50 °C; (**e**) POD et 35 °C and (**f**) POD at 50 °C.

The initial ζ-potential value of native *α*-amylase dissolved in ultrapure water was −25 mV (Figure 5a,b), which agrees well with the previously published results of Li et al. [57]. After 1 h exposure to supercritical conditions at 35 °C, an increase in the negative value to −34.4 mV was observed, while a statistically significant decrease in the initial ζ-potential to −20.1 mV was observed at a temperature of 50 °C. Further, at both temperatures, a pattern of an increase in the negative value appeared with prolongation of the treatment time. After 6 h no statistically significant difference in ζ-potential was observed regardless of temperature (−37.5 mV and −39 mV at 35 °C and 50 °C, respectively).

In the case of lipase, an initial value of −11.9 mV was obtained for the ζ-potential in our study (Figure 5c,d). After exposure to the sc-CO_2_, the measured ζ-potential values were even more negative at both temperatures studied, with maximum values determined after 3 h of sc-CO_2_ treatment (−29.7 mV and −33.9 at 35 °C and 50 °C, respectively).

On the other hand, the results presented in Figure 5e,f show that the applied sc-CO_2_ treatment reduced the initial ζ-potential (−16.3 mV) of POD. For example, at a temperature of 35 °C, the lowest value (−5.87 mV) was observed after 3 h of exposure, whereas at a temperature of 50 °C, no statistically significant differences in the decrease in ζ-potential were observed regardless of the exposure time. This decrease in the ζ-potential of POD agrees well with the particle size distribution results, suggesting that the decrease in negative charge on the molecular surfaces leads to aggregation of the molecules and consequently to a loss of the original enzyme activity.

Based on the some previously described theories, zeta potential of the enzymes under the supercritical conditions can also be caused by changes in the pH value [38]. According to the theory, when the pH of the environment falls to the isoelectric point of the enzyme, the negative charge of the molecules decreases, which leads to aggregation of the enzyme molecules [66]. Indeed, the reduction in ζ-potential weakened the electrostatic repulsion between molecules, which favoured the process of enzyme aggregation [64]. On the other hand, the reduction in the negative charge on the enzyme surface under the influence of sc-CO_2_ can probably be explained by conformational changes in the secondary structure of the enzyme caused by the migration of some negatively/positively charged amino acid residues to the surface of the enzyme, which ultimately led to a change in the ζ-potential.

## 4. Conclusions

The results of our study suggest that sc-CO_2_ technology may be a promising technology that can be used effectively in processes where controlled enzyme activity is required. By monitoring the activity of native enzymes in the solid state after exposure to supercritical conditions, we concluded that the changes in the structure of the enzyme itself caused by treatment parameters are crucial for enzyme activity. Further, it was demonstrated, how the same treatment parameters differently influence the secondary structure of different enzymes. In our study, α-amylase showed the highest resistance to structural changes under the conditions tested, while peroxidase showed a significant percentage of inactivation. It was shown how the changes in the secondary structures of the enzymes were responsible for these changes in enzyme activities. On the other hand, however, there were significant deviations in the expected values of ζ-potential and particle size distribution as a consequence of the enzyme treatment parameters. Therefore, it can probably be concluded that the mechanism of action of sc-CO_2_ on the enzyme in the solid state is not subject to the same changes in the tertiary structure of the protein as when the enzymes are treated with other technologies. To confirm this theory, further studies on the changes in enzyme conformations after sc-CO_2_ treatment are required.

## Figures and Tables

**Table 1:** The effect of sc-CO_2_ treatment under different conditions on total protein content and residual enzyme activities in white wheat flour. ^a,b,c,d,e^ Different superscripts in the same line indicate significant differences between the values obtained at the 95% confidence level (*p* < 0.05) according to the Student-Newman-Keuls (S-N-K) test. The results are given as mean ± standard deviation.

Determined Values		Control Sample (Untreated)	Temperature					
			35 °C		42.5 °C		50 °C	
			Pressure (Bar)					
			200	300	200	300	200	300
Total protein content (g/100g)		1.23 ± 0.02 ^a^	1.23 ± 0.01 ^a^	1.22 ± 0.04 ^a^	1.24 ± 0.03 ^a^	1.22 ± 0.03 ^a^	1.33 ± 0.00 ^a^	1.28 ± 0.08 ^a^
Residual activity (%)	α-amylase	100 ^a^	115.4 ± 10.9 ^a^	115.2 ± 10.2 ^a^	125.6 ± 16.6 ^a^	116.0 ± 8.2 ^a^	114.1 ± 13.0 ^a^	112.1 ± 4.6 ^a^
	lipase	100 ^e^	38.5 ± 2.7 ^b,c^	60.5 ± 3.0 ^d^	93.5 ± 8.6 ^e^	42.5 ± 3.9 ^c^	29.7 ± 2.7 ^b^	18.4 ± 1.2 ^a^
	POD	100 ^a^	124.5 ± 1.0 ^b^	135.0 ± 2.5 ^c^	105.6 ± 4.8 ^a^	101.6 ± 5.2 ^a^	105.9 ± 2.4 ^a^	103.0 ± 1.7 ^a^

## Data Availability

Data are contained within the article and Supplementary Material.

## Supplementary Materials

The following supporting information can be downloaded at: https://www.mdpi.com/article/10.3390/foods12244499/s1. Figure S1: ζ-potential determination of α-amylase before and after 1h, 3h and 6h sc-CO_2_ treatment at two different temperatures (35 °C and 50 °C); Figure S2: ζ-potential determination of lipase before and after 1h, 3h and 6h sc-CO_2_ treatment at two different temperatures (35 °C and 50 °C); Figure S3: ζ-potential determination of POD before and after 1h, 3h and 6h sc-CO_2_ treatment at a temperature of 35 °C.

## References

[1] Shouket S., Khurshid S., Khan J., Batool R., Sarwar A., Aziz T., Alhomrani M., Alamri A.S., Sameeh M.Y., Zubair Filimban F. (2023). Enhancement of shelf-life of food items via immobilized enzyme nanoparticles on varied supports. A sustainable approach towards food safety and sustainability. Food Res. Int..

[2] Leitgeb M., Knez Ž., Hojnik Podrepšek G. (2022). Effect of Green Food Processing Technology on the Enzyme Activity in Spelt Flour. Foods.

[3] Li M.J., Wang H.R., Tong L.T., Fan B., Yang X.J., Sun R.Q., Liu L.Y., Wang F.Z., Wang L.L. (2022). A comparison study of three heating assisted enzyme inactivation pretreatments on the physicochemical properties and edible quality of highland barley grain and flour. J. Cereal Sci..

[4] Lancelot E., Fontaine J., Grua-Priol J., Le-Bail A. (2021). Effect of long-term storage conditions on wheat flour and bread baking properties. Food Chem..

[5] Gu Y., Qian X., Sun B., Tian X., Wang X., Ma S. (2022). Effect of roasting treatment on the micromorphology, gelatinization, structure, and digestibility of whole oat flour. LWT.

[6] Zhao B., Shang J., Liu L., Tong L.T., Zhou X., Wang S., Zhang Y., Wang L., Zhou S. (2020). Effect of roasting process on enzymes inactivation and starch properties of highland barley. Int. J. Biol. Macromol..

[7] Bulhões Bezerra Cavalcante T.A., dos Santos Funcia E., Wilhelms Gut J.A. (2021). Inactivation of polyphenol oxidase by microwave and conventional heating: Investigation of thermal and non-thermal effects of focused microwaves. Food Chem..

[8] Kubo M.T.K., Curet S., Augusto P.E.D., Boillereaux L. (2019). Multiphysics modeling of microwave processing for enzyme inactivation in fruit juices. J. Food Eng..

[9] Hu Y., Wang L., Li Z. (2018). Superheated steam treatment on wheat bran: Enzymes inactivation and nutritional attributes retention. LWT.

[10] Wu Y., Li W., Martin G.J.O., Ashokkumar M. (2021). Mechanism of low-frequency and high-frequency ultrasound-induced inactivation of soy trypsin inhibitors. Food Chem..

[11] Yu Z.L., Zeng W.C., Zhang W.H., Liao X.P., Shi B. (2014). Effect of ultrasound on the activity and conformation of α-amylase, papain and pepsin. Ultrason. Sonochem..

[12] Yao Y., Zhang B., Pang H., Wang Y., Fu H., Chen X., Wang Y. (2022). The effect of radio frequency heating on the inactivation and structure of horseradish peroxidase. Food Chem..

[13] Xu J., Wang B., Wang Y., Zhang M., Chitrakar B. (2020). Effect of Applied Voltage on the Aggregation and Conformational Changes in Peroxidase Under Electrospray. Food Bioprocess. Technol..

[14] Guionet A., Fujiwara T., Sato H., Takahashi K., Takaki K., Matsui M., Tanino T., Ohshima T. (2021). Pulsed electric fields act on tryptophan to inactivate α-amylase. J. Electrostat..

[15] Gu Y., Shi W., Liu R., Xing Y., Yu X., Jiang H. (2021). Cold plasma enzyme inactivation on dielectric properties and freshness quality in bananas. Innov. Food Sci. Emerg. Technol..

[16] Marszałek K., Doesburg P., Starzonek S., Szczepańska J., Woźniak Ł., Lorenzo J.M., Skaopska S., Rzoska S., Barba F.J. (2019). Comparative effect of supercritical carbon dioxide and high pressure processing on structural changes and activity loss of oxidoreductive enzymes. J. CO2 Util..

[17] Marszałek K., Kruszewski B., Woźniak Ł., Skąpska S. (2017). The application of supercritical carbon dioxide for the stabilization of native and commercial polyphenol oxidases and peroxidases in cloudy apple juice (cv. Golden Delicious). Innov. Food Sci. Emerg. Technol..

[18] Monhemi H., Jalali B. (2021). Changing the residues interaction pattern as a universal mechanism for enzyme inactivation and denaturation in supercritical CO_2_. J. Mol. Liq..

[19] Zambon A., Facco P., Morbiato G., Toffoletto M., Poloniato G., Sut S., Andrigo P., Dall’Acqua S., de Bernard M., Spilimbergo S. (2022). Promoting the preservation of strawberry by supercritical CO2 drying. Food Chem..

[20] Suo T., Guo X.N., Zhu K.X. (2022). Effects of tempering with plasma-activated water on total plate count and quality properties of wheat flour. J. Cereal Sci..

[21] Zeng Z., Wang Y., Xu G., Zhou L., Liu C., Luo S. (2023). Peroxidase inactivation by cold plasma and its effects on the storage, physicochemical and bioactive properties of brown rice. Food Biosci..

[22] Podrepšek G.H., Knez Ž., Leitgeb M. (2020). Development of chitosan functionalized magnetic nanoparticles with bioactive compounds. Nanomaterials.

[23] Leitgeb M., Knez Ž., Podrepšek G.H. (2022). Enzyme Activity and Physiochemical Properties of Flour after Supercritical Carbon Dioxide Processing. Foods.

[24] Bertolini F.M., Morbiato G., Facco P., Marszałek K., Pérez-Esteve É., Benedito J., Zambon A., Spilimbergo S. (2020). Optimization of the supercritical CO_2_ pasteurization process for the preservation of high nutritional value of pomegranate juice. J. Supercrit. Fluids.

[25] Machado N.D., Mosquera J.E., Martini R.E., Goñi M.L., Gañán N.A. (2022). Supercritical CO_2_-assisted impregnation/deposition of polymeric materials with pharmaceutical, nutraceutical, and biomedical applications: A review (2015–2021). J. Supercrit. Fluids.

[26] Pravallika K., Chakraborty S., Singhal R.S. (2023). Supercritical drying of food products: An insightful review. J. Food Eng..

[27] Zorić M., Banožić M., Aladić K., Vladimir-Knežević S., Jokić S. (2022). Supercritical CO_2_ extracts in cosmetic industry: Current status and future perspectives. Sustain. Chem. Pharm..

[28] Monhemi H. (2021). Protein simulation in supercritical CO2: The challenge of force field. J. Mol. Liq..

[29] Sheikh M.A., Saini C.S., Sharma H.K. (2023). Structural modification of plum (*Prunus domestica* L) kernel protein isolate by supercritical carbon-dioxide treatment: Functional properties and in-vitro protein digestibility. Int. J. Biol. Macromol..

[30] Gui F., Chen F., Wu J., Wang Z., Liao X., Hu X. (2006). Inactivation and structural change of horseradish peroxidase treated with supercritical carbon dioxide. Food Chem..

[31] Marszałek K., Krzyżanowska J., Woźniak Ł., Skąpska S. (2017). Kinetic modelling of polyphenol oxidase, peroxidase, pectin esterase, polygalacturonase, degradation of the main pigments and polyphenols in beetroot juice during high pressure carbon dioxide treatment. LWT.

[32] Marszałek K., Skąpska S., Woźniak Ł., Sokołowska B. (2015). Application of supercritical carbon dioxide for the preservation of strawberry juice: Microbial and physicochemical quality, enzymatic activity and the degradation kinetics of anthocyanins during storage. Innov. Food Sci. Emerg. Technol..

[33] Leitgeb M., Čolnik M., Primožič M., Zalar P., Cimerman N.G., Knez Ž. (2013). Activity of cellulase and α-amylase from Hortaea werneckii after cell treatment with supercritical carbon dioxide. J. Supercrit. Fluids.

[34] Senyay-Oncel D., Yesil-Celiktas O. (2011). Activity and stability enhancement of α-amylase treated with sub- and supercritical carbon dioxide. J. Biosci. Bioeng..

[35] Dos Santos P., Rezende C.A., Martínez J. (2016). Activity of immobilized lipase from Candida antarctica (Lipozyme 435) and its performance on the esterification of oleic acid in supercritical carbon dioxide. J. Supercrit. Fluids.

[36] Podrepšek G.H., Knez Ž., Leitgeb M. (2020). The Influence of Supercritical Carbon Dioxide on Graham Flour Enzyme Polyphenol Oxidase Activity. Molecules.

[37] Pliego J., Mateos J.C., Rodriguez J., Valero F., Baeza M., Femat R., Camacho R., Sandoval G., Herrera-López E.J. (2015). Monitoring lipase/esterase activity by stopped flow in a sequential injection analysis system using p-nitrophenyl butyrate. Sensors.

[38] Li J., Zhu L., Murtaza A., Iqbal A., Zhang J., Xu X., Pan S., Hu W. (2022). The effect of high pressure carbon dioxide on the inactivation kinetics and structural alteration of phenylalanine ammonia-lyase from Chinese water chestnut: An investigation using multi-spectroscopy and molecular docking methods. Innov. Food Sci. Emerg. Technol..

[39] Allai F.M., Azad Z.R.A.A., Mir N.A., Gul K. (2023). Recent advances in non-thermal processing technologies for enhancing shelf life and improving food safety. Appl. Food Res..

[40] Feng J., Zheng Y., Zhang X., Zhou R., Ma M. (2023). Effect of supercritical carbon dioxide on bacterial community, volatile profiles and quality changes during storage of Mongolian cheese. Food Control..

[41] Zhang L., Liu S., Ji H., Zhang C., Deng C., Cao W., Mao W., Gao J. (2011). Inactivation of polyphenol oxidase from Pacific white shrimp by dense phase carbon dioxide. Innov. Food Sci. Emerg. Technol..

[42] Bahrami N., Bayliss D., Chope G., Penson S., Perehinec T., Fisk I.D. (2016). Cold plasma: A new technology to modify wheat flour functionality. Food Chem..

[43] Yadav J.K., Prakash V. (2009). Thermal stability of α-amylase in aqueous cosolvent systems. J. Biosci..

[44] De Schepper C.F., Buvé C., Van Loey A.M., Courtin C.M. (2022). A kinetic study on the thermal inactivation of barley malt α-amylase and β-amylase during the mashing process. Food Res. Int..

[45] Mathias O.K., Michael D., Forbes L., Joseph A. (2010). Lipase inactivation by pressurized CO2 in presence of sensitizing additives. Eur. J. Sci. Res..

[46] Ling B., Lyng J.G., Wang S. (2018). Effects of hot air-assisted radio frequency heating on enzyme inactivation, lipid stability and product quality of rice bran. LWT.

[47] Skřivan P., Sluková M., Jurkaninová L., Švec I. (2021). Preliminary investigations on the use of a new milling technology for obtaining wholemeal flours. Appl. Sci..

[48] Leitgeb M., Knez Z. (1990). The influence of water on the synthesis of n-butyl oleate by immobilized Mucor miehei lipase. J. Am. Oil Chem. Soc..

[49] Hu W., Zhou L., Xu Z., Zhang Y., Liao X. (2013). Enzyme Inactivation in Food Processing using High Pressure Carbon Dioxide Technology. Crit. Rev. Food Sci. Nutr..

[50] Amaral G.V., Silva E.K., Cavalcanti R.N., Cappato L.P., Guimaraes J.T., Alvarenga V.O., Esmerino E.A., Portela J.B., Sant’ Ana A.S., Freitas M.Q. (2017). Dairy processing using supercritical carbon dioxide technology: Theoretical fundamentals, quality and safety aspects. Trends Food Sci. Technol..

[51] Kamat S., Critchley G., Beckman E.J., Russell A.J. (1995). Biocatalytic Synthesis of Acrylates in Organic Solvents and Supercritical Fluids: 111. Does Carbon Dioxide Covalently Modify Enzymes? yanjay. Biotechnol. Bioeng..

[52] Habulin M., Knez Ž. (1993). Influence of Reaction Parameters on Synthesis of n-Butyl Oleate by. Eur. J. Lipid Sci. Technol..

[53] Wimmer Z., Zarevúcka M. (2010). A review on the effects of supercritical carbon dioxide on enzyme activity. Int. J. Mol. Sci..

[54] Han Y.X., Cheng J.H., Sun D.W. (2019). Changes in activity, structure and morphology of horseradish peroxidase induced by cold plasma. Food Chem..

[55] Miles A.J., Janes R.W., Wallace B.A. (2021). Tools and methods for circular dichroism spectroscopy of proteins: A tutorial review. Chem. Soc. Rev..

[56] Greenfield N.J. (2007). Using circular dichroism spectra to estimate protein secondary structure. Nat. Protoc..

[57] Li J., Zhang J., Li C., Huang W., Guo C., Jin W., Shen W. (2022). Structural Transitions of Alpha-Amylase Treated with Pulsed Electric Fields: Effect of Coexisting Carrageenan. Foods.

[58] Tian M.L., Fang T., Du M.Y., Zhang F.S. (2016). Effects of Pulsed Electric Field (PEF) Treatment on Enhancing Activity and Conformation of α-Amylase. Protein J..

[59] Esmaeilnejad-Ahranjani P., Kazemeini M., Singh G., Arpanaei A. (2015). Amine-functionalized magnetic nanocomposite particles for efficient immobilization of lipase: Effects of functional molecule size on properties of the immobilized lipase. RSC Adv..

[60] Kumar V., Yedavalli P., Gupta V., Rao N.M. (2014). Engineering lipase A from mesophilic Bacillus subtilis for activity at low temperatures. Protein Eng. Des. Sel..

[61] Shikha S., Thakur K.G., Bhattacharyya M.S. (2017). Facile fabrication of lipase to amine functionalized gold nanoparticles to enhance stability and activity. RSC Adv..

[62] Guo Y., Wu B., Guo X., Liu D., Qiu C., Ma H. (2022). Thermosonication inactivation of horseradish peroxidase with different frequency modes: Effect on activity, structure, morphology and mechanisms. Food Chem..

[63] Li F., Tang Y. (2021). The activation mechanism of peroxidase by ultrasound. Ultrason. Sonochem..

[64] Hu W., Zhang Y., Wang Y., Zhou L., Leng X., Liao X., Hu X. (2011). Aggregation and homogenization, surface charge and structural change, and inactivation of mushroom tyrosinase in an aqueous system by subcritical/supercritical carbon dioxide. Langmuir.

[65] Nadar S.S., Rathod V.K. (2017). Sonochemical Effect on Activity and Conformation of Commercial Lipases. Appl. Biochem. Biotechnol..

[66] Jachimska B., Wasilewska M., Adamczyk Z. (2008). Characterization of globular protein solutions by dynamic light scattering, electrophoretic mobility, and viscosity measurements. Langmuir.

