# Supplementary material for: Influence of Supercritical Carbon Dioxide on the Activity and Conformational Changes of α-Amylase, Lipase, and Peroxidase in the Solid State Using White Wheat Flour as an Example
Source: Foods. 2023 Dec 16;12(24):4499. doi: 10.3390/foods12244499 (PMC10743174; doi:10.3390/foods12244499)
Supplement: Supplementary file 1 [file foods-12-04499-s001.zip › foods-2764292-supplementary.pdf]

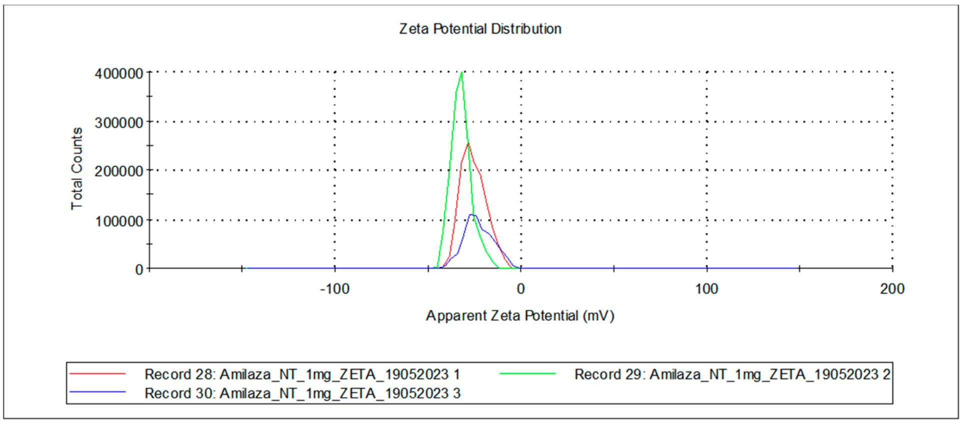
Zeta Potential Distribution
Total Counts
400000
300000
200000
100000
0
-100
0
100
200
Apparent Zeta Potential (mV)
Record 28: Amilaza_NT_1mg_ZETA_19052023 1
Record 29: Amilaza_NT_1mg_ZETA_19052023 2
Record 30: Amilaza_NT_1mg_ZETA_19052023 3

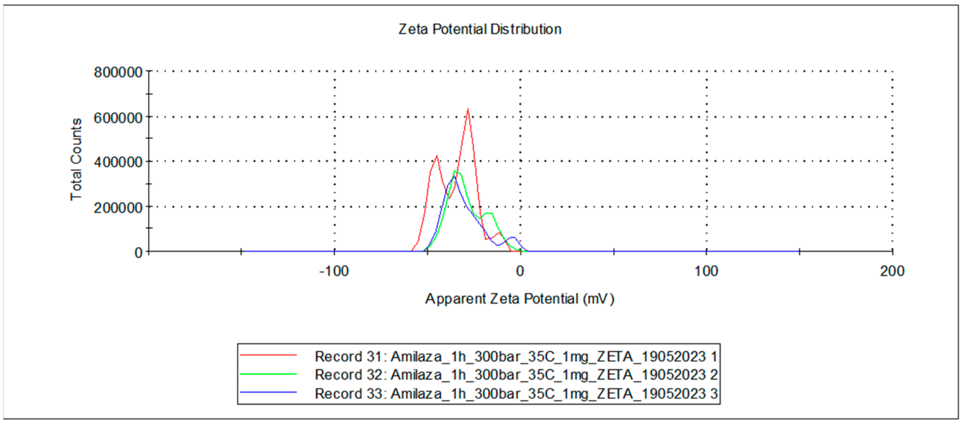
Zeta Potential Distribution
Total Counts
800000
600000
400000
200000
0
-100
0
100
200
Apparent Zeta Potential (mV)
Record 31: Amilaza_1h_300bar_35C_1mg_ZETA_19052023 1
Record 32: Amilaza_1h_300bar_35C_1mg_ZETA_19052023 2
Record 33: Amilaza_1h_300bar_35C_1mg_ZETA_19052023 3

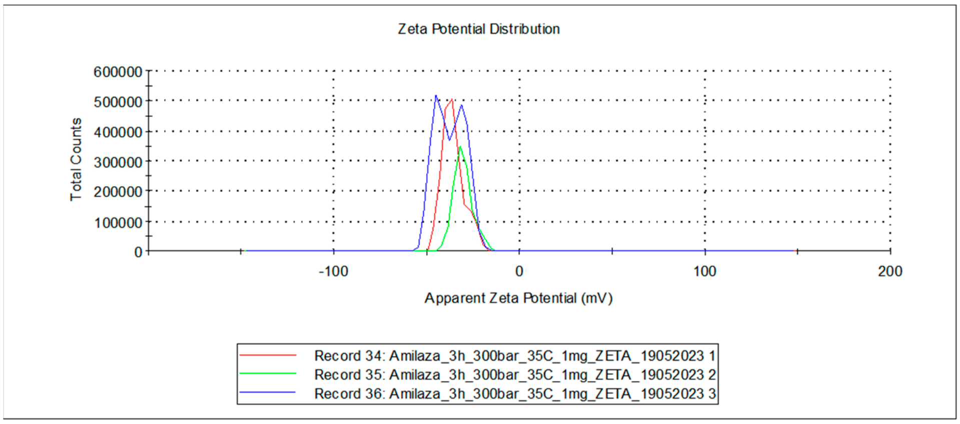
Zeta Potential Distribution
Total Counts
600000
500000
400000
300000
200000
100000
0
-100
0
100
200
Apparent Zeta Potential (mV)
Record 34: Amilaza_3h_300bar_35C_1mg_ZETA_19052023 1
Record 35: Amilaza_3h_300bar_35C_1mg_ZETA_19052023 2
Record 36: Amilaza_3h_300bar_35C_1mg_ZETA_19052023 3

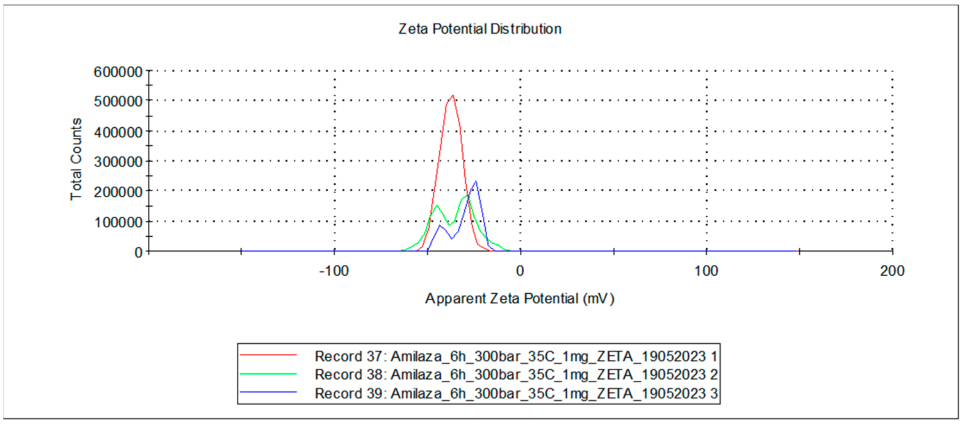
Zeta Potential Distribution
Total Counts
600000
500000
400000
300000
200000
100000
0
-100
0
100
200
Apparent Zeta Potential (mV)
Record 37: Amilaza_6h_300bar_35C_1mg_ZETA_19052023 1
Record 38: Amilaza_6h_300bar_35C_1mg_ZETA_19052023 2
Record 39: Amilaza_6h_300bar_35C_1mg_ZETA_19052023 3

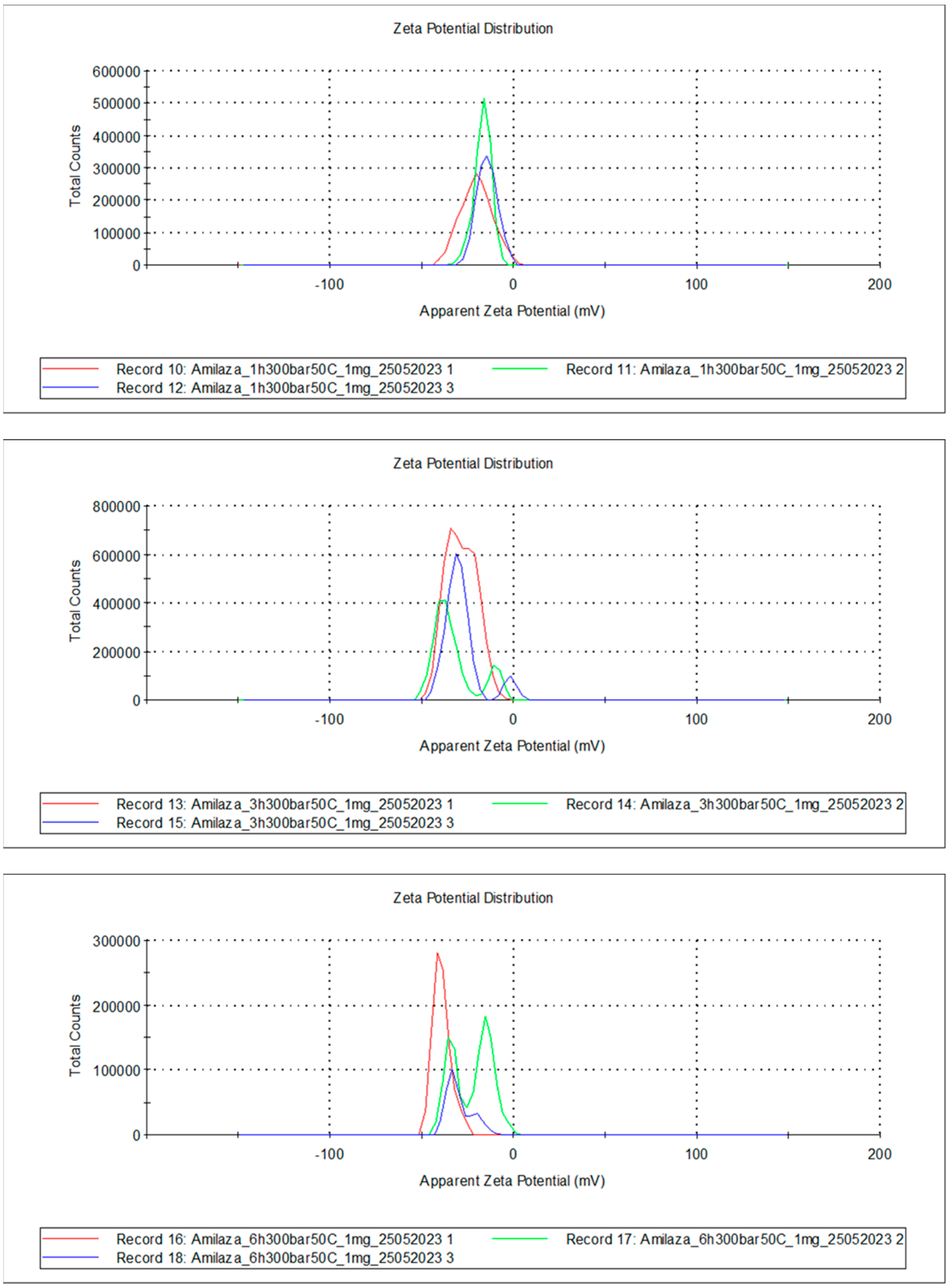

Figure S1. ζ-potential determination of α-amylase before and after 1h, 3h and 6h sc-CO_2 treatment at two different temperatures (35°C and 50°C).

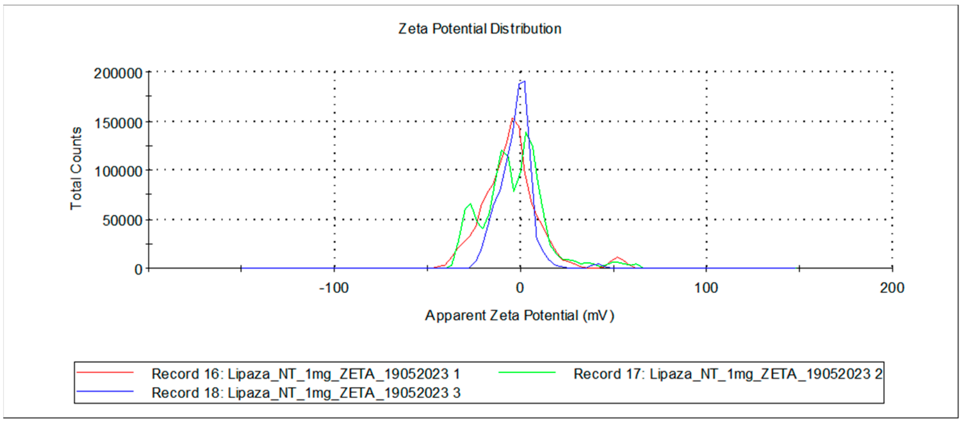
Zeta Potential Distribution
Total Counts
200000
150000
100000
50000
0
-100
0
100
200
Apparent Zeta Potential (mV)
Record 16: Lipaza_NT_1mg_ZETA_19052023 1
Record 17: Lipaza_NT_1mg_ZETA_19052023 2
Record 18: Lipaza_NT_1mg_ZETA_19052023 3

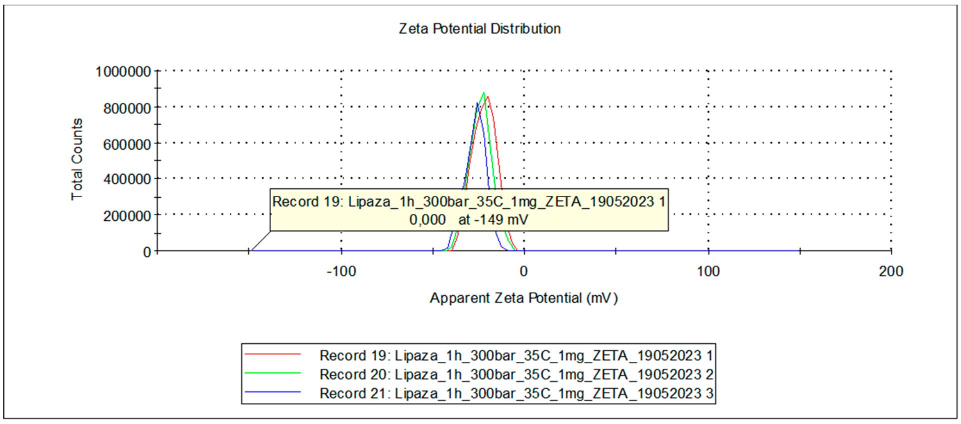
Zeta Potential Distribution
Total Counts
1000000
800000
600000
400000
200000
0
Record 19: Lipaza_1h_300bar_35C_1mg_ZETA_19052023 1
0,000 at -149 mV
-100
0
100
200
Apparent Zeta Potential (mV)
Record 19: Lipaza_1h_300bar_35C_1mg_ZETA_19052023 1
Record 20: Lipaza_1h_300bar_35C_1mg_ZETA_19052023 2
Record 21: Lipaza_1h_300bar_35C_1mg_ZETA_19052023 3

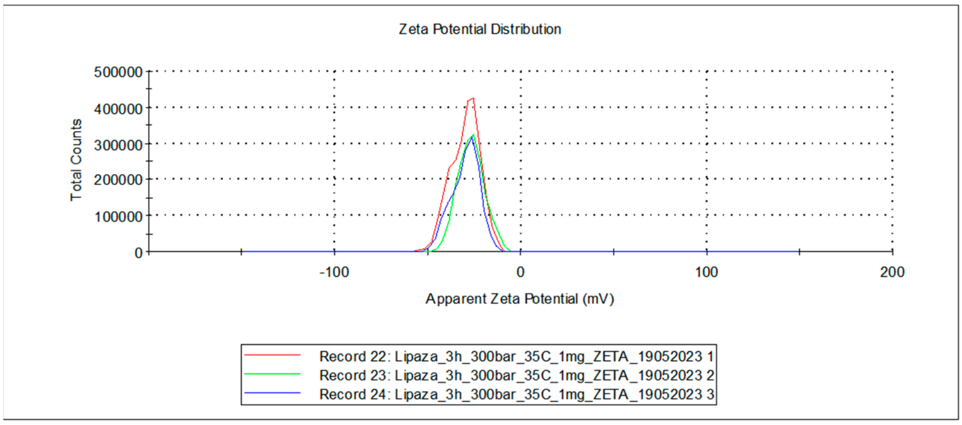
Zeta Potential Distribution
Total Counts
500000
400000
300000
200000
100000
0
-100
0
100
200
Apparent Zeta Potential (mV)
Record 22: Lipaza_3h_300bar_35C_1mg_ZETA_19052023 1
Record 23: Lipaza_3h_300bar_35C_1mg_ZETA_19052023 2
Record 24: Lipaza_3h_300bar_35C_1mg_ZETA_19052023 3

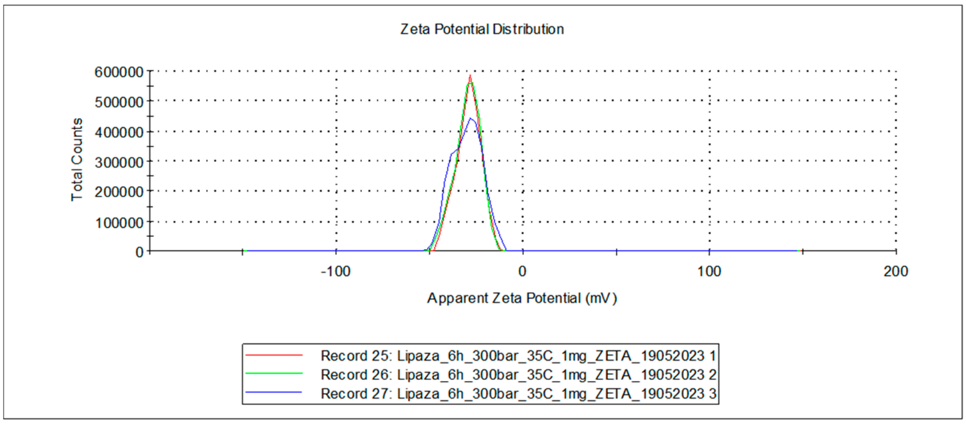
Zeta Potential Distribution
Total Counts
600000
500000
400000
300000
200000
100000
0
-100
0
100
200
Apparent Zeta Potential (mV)
Record 25: Lipaza_6h_300bar_35C_1mg_ZETA_19052023 1
Record 26: Lipaza_6h_300bar_35C_1mg_ZETA_19052023 2
Record 27: Lipaza_6h_300bar_35C_1mg_ZETA_19052023 3

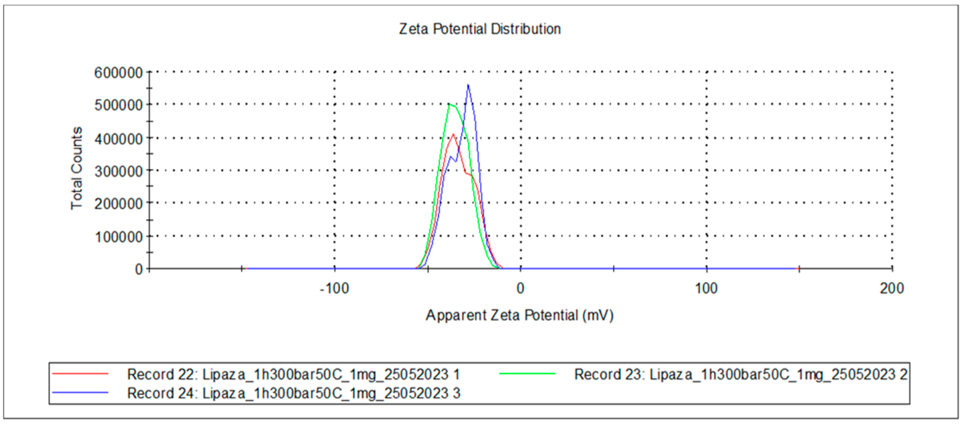

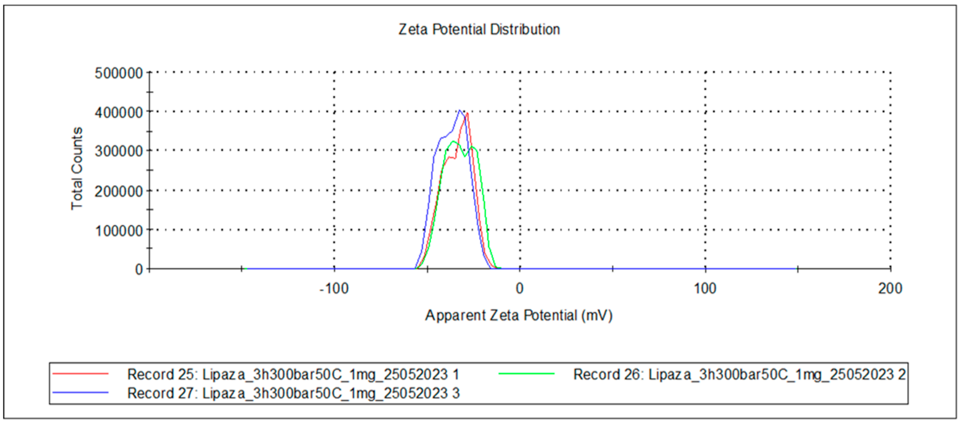

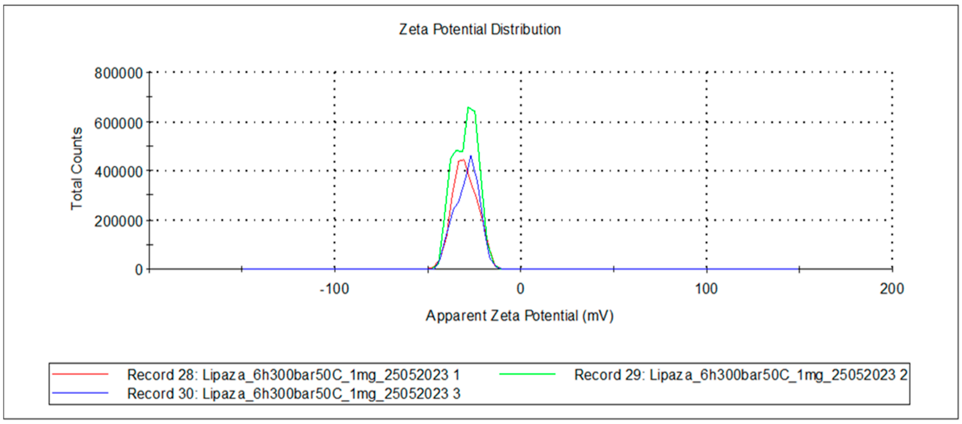

Figure S2. ζ-potential determination of lipase before and after 1h, 3h and 6h sc-CO_2 treatment at two different temperatures (35°C and 50°C).

.

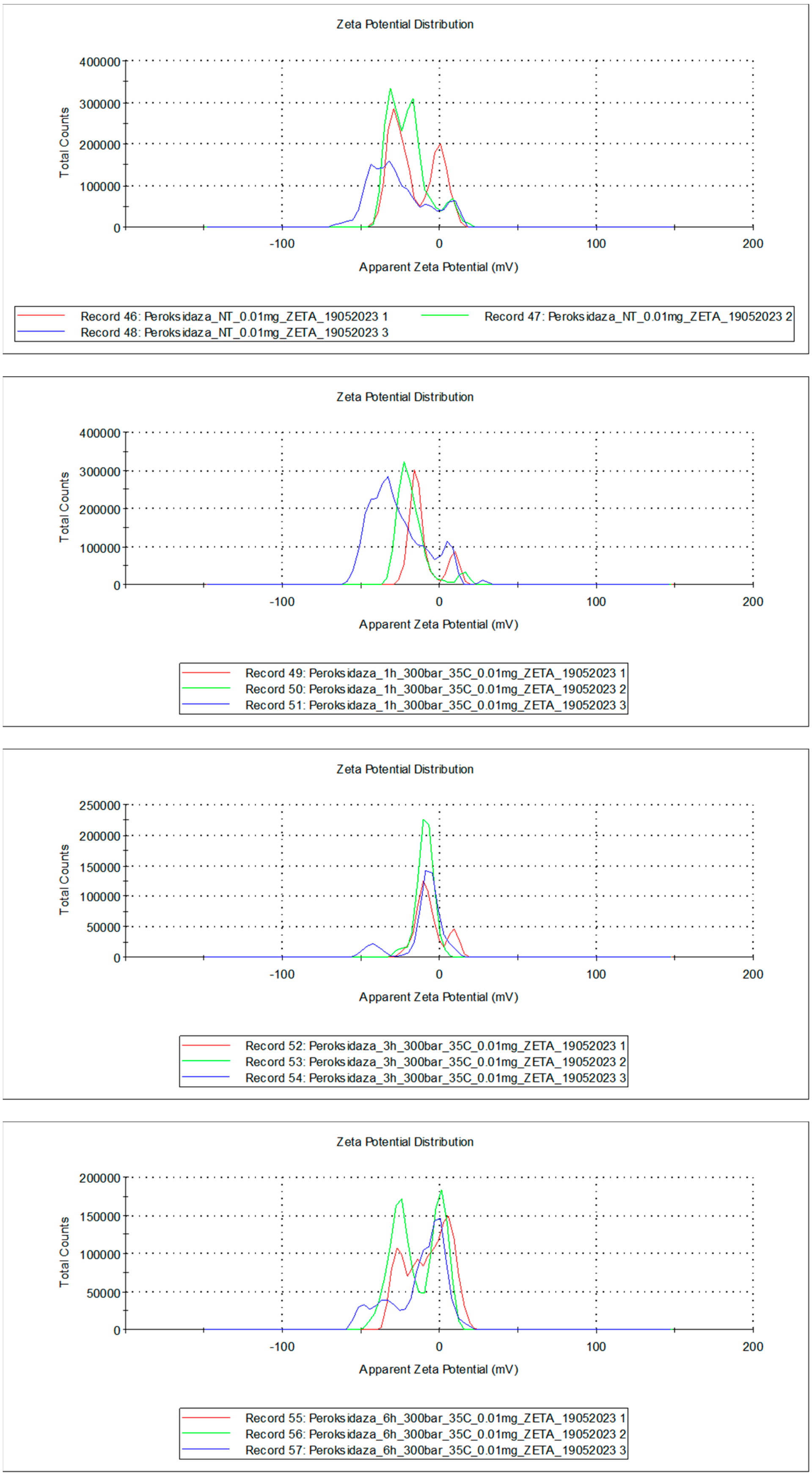

Figure S3. ζ-potential determination of POD before and after 1h, 3h and 6h sc-CO_2 treatment at a temperature of 35°C.
